# Hidden Markov model speed heuristic and iterative HMM search procedure

**DOI:** 10.1186/1471-2105-11-431

**Published:** 2010-08-18

**Authors:** L Steven Johnson, Sean R Eddy, Elon Portugaly

**Affiliations:** 1 Department of Immunology and Pathology, Washington University School of Medicine, St. Louis, Missouri, USA; 2 Janelia Farm Research Campus, Howard Hughes Medical Institute, Ashburn, Virginia, USA; 3 School of Computer Science & Engineering, The Hebrew University of Jerusalem, Jerusalem, Israel

## Abstract

**Background:**

Profile hidden Markov models (profile-HMMs) are sensitive tools for remote protein homology detection, but the main scoring algorithms, Viterbi or Forward, require considerable time to search large sequence databases.

**Results:**

We have designed a series of database filtering steps, HMMERHEAD, that are applied prior to the scoring algorithms, as implemented in the HMMER package, in an effort to reduce search time. Using this heuristic, we obtain a 20-fold decrease in Forward and a 6-fold decrease in Viterbi search time with a minimal loss in sensitivity relative to the unfiltered approaches. We then implemented an iterative profile-HMM search method, JackHMMER, which employs the HMMERHEAD heuristic. Due to our search heuristic, we eliminated the subdatabase creation that is common in current iterative profile-HMM approaches. On our benchmark, JackHMMER detects 14% more remote protein homologs than SAM's iterative method T2K.

**Conclusions:**

Our search heuristic, HMMERHEAD, significantly reduces the time needed to score a profile-HMM against large sequence databases. This search heuristic allowed us to implement an iterative profile-HMM search method, JackHMMER, which detects significantly more remote protein homologs than SAM's T2K and NCBI's PSI-BLAST.

## Background

Profile hidden Markov models (profile-HMMs) are sensitive tools for remote protein homology detection. Unfortunately, current implementations of the Viterbi and Forward scoring algorithms, which are commonly used to compare a sequence to a profile-HMM, require considerable time. Scoring an average length profile-HMM against the NCBI NR database (See Methods section.) using the Forward algorithm takes approximately 5 hours on a 2.66 Ghz desktop using HMMER [1]. In comparison, the heuristic pairwise sequence comparison method WU-BLAST is greater than 200-fold faster [2,3]. WU-BLAST achieves this speed, in part, by using several incremental steps to determine whether a database sequence is sufficiently similar to the query. If a database sequence fails at any of these stages, the sequence comparison is halted and the next database sequence is examined. This fast elimination of the majority of database sequences contributes to the significant reduction in search time [2,3].

Such sequence-level filtering can be easily and simply combined with existing profile hidden Markov model search strategies. In this study, we describe a series of filtering steps that are applied prior to the Viterbi and Forward profile-HMM scoring algorithms as implemented in an exploratory branch of the HMMER2 software package, HMMER 2.5.1. These filtering steps were selected with the goal of reducing profile-HMM search time against large sequence databases while minimizing the reduction in remote homology detection.

This scoring heuristic was then utilized in an iterative profile hidden Markov model search method. Iterative search methods perform multiple searches of a sequence database. Once novel homologous sequences are identified, they are incorporated into the statistical model of the query sequence. This new model is then re-searched against the sequence database. Iterative search approaches have been utilized for sequence to position-specific scoring matrix comparison methods, e.g. PSI-BLAST, as well as profile hidden Markov model to sequence scoring, e.g. SAM's target2k [3-6]. At the core of these methods is a probabilistic model of the query protein built from multiple homologous sequences. One major factor in the sensitivity of these methods is how well these homologous sequences represent the diversity of the protein family being modelled. In principle, by incorporating distant homologous sequences, while avoiding contaminating non-homologous sequences, into the model after each search, iterative methods are able to build a more diverse and potentially more sensitive model of the query protein.

Current iterative methods for profile hidden Markov models are dependent on creating a subdatabase from a larger sequence database using a less sensitive pairwise sequence comparison method, such as WU-BLAST. This subdatabase is then used in the iterative searches due to the speed issues inherent in using profile-HMM scoring algorithms on large databases [4,5]. However, using a less sensitive pairwise sequence method in the creation of the subdatabase presents a potential limitation to current iterative profile-HMM methods. By taking advantage of our newly developed search heuristic, we have eliminated the requirement of this subdatabase. In order to determine any sensitivity loss by our search heuristic and to measure the performance of our iterative search method, we have created a remote protein homology benchmark.

## Methods

### Non-Redundant NCBI NR database, NRDB90

The non-redundant database, NRDB90, used in this study was created from NCBI's NR, 1/12/06, database which was filtered to remove sequences with greater than 90% identity using Holm and Sander's method [7]. The script used is available at: http://ekhidna.biocenter.helsinki.fi/downloads/rsdb/nrdb90.pl.

### Remote Protein Homology Benchmark

One crucial aspect of a remote protein homology benchmark is a set of trusted protein homologs. Current datasets of homologous proteins primarily utilize either protein structure or sequence conservation to infer homology [8-10]. Since using a sequence-based protein homolog dataset to test sequence-based homology detection algorithms could be a source of confounding circularity, we chose to use the structure-based SCOP database as a trusted source of true homologs. It should be noted that this database consists of single domain proteins that are relatively less compositionally biased and repetitive than those in the large sequence repositories. Similar to several other studies, we used the Astral Compendium version of the SCOP 1.5 database filtered for 40% identity [5,11-13].

In order to generate multiple sequence alignments for each test sequence, we utilized an approach similar to that used by Madera and Gough [13]. Each test sequence was searched against a non-redundant version of NCBI's NR database, NRDB90, using WU-BLAST blastp 2.0 MP-WashU [7]. Those NRDB90 sequences that matched the test sequence with an E-value less than 1 × 10^-3 ^were aligned, with the test sequence, using ClustalW 1.82. These multiple sequence alignments were then used in the construction and calibration of profile hidden Markov models using HMMER 2.5.1 (see Methods) and SAM 3.5 in the HMMERHEAD benchmarking studies. However, in benchmarking the iterative methods, each program was given the individual test sequence and allowed to iteratively search NRDB90 to build a model.

Almost as crucial as a set of true homologs in a remote protein homology benchmark is the set of true non-homologs. A common utilization of the SCOP classification system in a protein homology benchmark is to declare that those proteins that share the same SCOP Superfamily classification are homologs [5,13]. Those proteins that belong to different SCOP Fold classifications are considered non-homologous.

We, as well as others, have observed complications with using the SCOP classification scheme to distinguish non-homologous proteins [5,13]. Using several different sequence comparison methods, we observed highly significant scores between specific models and sequences with different SCOP Folds. Later versions of the SCOP database reclassified these sequences as being in the same Superfamily. This raises the important issue that one can not be positive two sequences are not homologous just because of their current SCOP Fold classification. In previous studies, the authors excluded hits between certain Folds from their benchmarking results due to the similarity and high level of cross-Fold hits they observed. Instead of selectively excluding hits between certain Folds, we decided to utilize shuffled SCOP sequences as a trusted source of non-homologous sequences. The program shuffle, from the HMMER 2.5.1 SQUID library, was used to create five different shuffled copies of each Astral sequence.

Under our benchmarking scheme, those proteins that share the same SCOP Superfamily classification will be considered a true homologous relationship. Those SCOP Superfamilies that consisted of one sequence were discarded from our benchmark. Any hit between a model and a shuffled SCOP sequence is considered a false homologous relationship and a hit between SCOP sequences not of the same Superfamily are considered ambiguous. Given the above design and homology criteria, we have produced a benchmark of 2,521 query alignments and a test database of 16,986 sequences. By comparing each query to the test database, there exists a total of 39,733 true homologous pairwise relationships and 36,466,265 non-homologous relationships.

### Sensitivity and Specificity Measures

The most common measures of sensitivity and specificity used in our studies was true positives identified versus errors per query. Errors per query, EPQ, is calculated by false positives divided by the number of search queries, 2521. Thus, the EPQ of 1, 3, 12, 25, 125, and 250 false positives are 0.0004, 0.001, 0.005, 0.01, 0.05, and 0.1.

For the studies examining HMMERHEAD parameter settings effects on performance we used minimum error rate to measure sensitivity and specificity in a combined metric [14]. We calculated minimum error rate as the minimum sum, over all possible choices of E-value threshold, of false positives and false negatives.

### Bootstrapping

In order to assess the significance of the performance differences between algorithms on our benchmark, we have utilized a bootstrapping approach [15]. We measure the performance of a program on 1000 replicate query sets which were generated by sampling the test database models with replacement. The performance of each method, e.g. HMMER or JackHMMER, was assessed for each query set. Therefore, for each query set we were able to calculate the 1000 values of TP_1_-TP_2_, where TP_1 _is the number of true positives identified at a given value of errors per query by method 1. e.g. HMMER. TP_2 _is the same value using method 2. e.g. JackHMMER. We then calculated a 95% confidence interval on the difference in true positives detected by the two different methods. This was accomplished by taking the 0.95 × 1000 = 950 middle values. If this distribution does not overlap zero, there is a statistical difference in the performance of these two methods.

### HMMER 2.5.1

HMMER 2.5.1 is an exploratory branch of the HMMER2 software where the major changes relevant to HMMER 2.3.2 are the incorporation of entropy weighting of sequences in the input alignment and the capability of calibrating a model's E-values using either Viterbi or Forward scoring. All models in this study were built using entropy weighting.

### HMMERHEAD Filtering Algorithm

HMMERHEAD's initial step is the generation and identification of significant "words". This step consists of identifying ungapped four residue words from a profile hidden Markov model's match state emission scores that possess a score above some threshold, θ. A word score is calculated as the sum of the log-odds emission scores of the word amino acid residues, as determined from the profile-HMM model. These words are then identified in the database sequences using a deterministic finite automaton.

In the next filtering step, each word identified in a database sequence is the seed for an ungapped alignment between the sequence and the profile-HMM. The alignment extension starts at the left end of the word and proceeds until the alignment score drops a threshold, δ/2, amount below the optimum score observed. Since this is an ungapped alignment, the alignment score is calculated as the sum of the match emission scores for the observed residues in the corresponding region of the profile-HMM. This extension is then performed on the opposite end of the word. Those extended-word alignments that possess a score above a threshold, μ, are passed on to the remaining filtering steps. Those database sequences that contain at least two qualifying extended-word hits are then examined further.

The final step consists of performing gapped Viterbi alignment between the sequence and the profile-HMM in the region between the two extended-word hits. If the Viterbi score is greater than the threshold, η, the sequence is then scored using HMMER's specified full scoring algorithm. i.e. Viterbi or Forward.

The HMMERHEAD search heuristic is accessible as a command line option in the HMMER 2.5.1 hmmsearch program. Execution of HMMER's hmmsearch program with the option "-h" will produce a full list of hmmsearch and HMMERHEAD command line options. For the HMMERHEAD benchmarking, models were built and calibrated from the benchmarking alignments using HMMER 2.5.1 hmmbuild and hmmcalibrate.

### JackHMMER

The JackHMMER procedure begins by identifying initial homologs to a single query sequence using either a default, or with user-defined parameters, NCBI or WU-BLAST database search. Subsequences of database targets identified with E-values less than 1 × 10^-3 ^are extracted and aligned using ClustalW. A hidden Markov model is built from this alignment and calibrated using HMMER. This model is then searched against a large sequence database, preferably filtered for fragmented and redundant sequences to decrease runtime, using HMMERHEAD Viterbi. Novel homologs are identified at each iteration and then aligned to the existing profile-HMM. The model is rebuilt and further searches are performed until no additional homologs are identified in the database or the maximum number of iterations has been performed.

The JackHMMER iterative search method is implemented as a stand-alone Perl script in HMMER 2.5.1. JackHMMER relies on several additional programs, such as either WU-BLAST or NCBI-BLAST, HMMER, and ClustalW. Paths to these programs are specified in the jhmmer_params file in the HMMER 2.5.1 src/directory and should be edited to the relevant locations of these programs in a user's file system. Execution of JackHMMER with no arguments will provide a full list of command line options.

JackHMMER performance in this study was determined using WU-BLAST and the non-redundant database NRDB90. Initial WU-BLAST hits with an E-value less than or equal to 1 × 10^-3 ^were aligned with the query using ClustalW. This multiple sequence alignment was used to build a HMMER Local/Local model using HMMER's hmmbuild, with default parameters, and calibrated using HMMER's hmmcalibrate, using 2000 random sequences. This profile-HMM was searched against NRDB90 using HMMERHEAD Viterbi with default parameters. The default E-value thresholds used in the iterative HMMERHEAD searches are [1 × 10^-5^, 1 × 10^-5^, 1 × 10^-4^, 3 × 10^-4^, 3 × 10^-4^, ...] for iterations 1 to the maximum number of iterations. The default maximum number of iterations is seven. The final alignment created from the JackHMMER iterative procedure was then used to build a local/local model, calibrated for Forward scoring, and searched against the test database.

### WU-BLAST and WU-BLAST FPS

The WU-BLAST searches used in the HMMERHEAD studies utilized WU-BLAST blastp 2.0 MP with default settings. WU-BLAST FPS used the Family Pairwise Search method [16]. This method consists of comparing a 'family' of sequences to a database using a pairwise sequence comparison method. In a Family Pairwise Search, when more than one 'family' sequence hits the same database sequence, their E-values are combined. We compared the performances of several methods of combining the E-values in this approach, such as mean log E-value, mean E-value, and minimum E-value. Since they provided the best performance on our benchmark, we used minimum E-values.

### NCBI PSI-BLAST

NCBI PSI-BLAST version 2.2.17 was used in this study. PSI-BLAST models were built by iteratively searching, using default settings, NRDB90 with the maximum number of iterations set to seven. The final PSI-BLAST model was then searched, using default settings, against the test database.

### SAM 3.5

SAM version 3.5 was used in this study. SAM's iterative search program target2k was searched against NRDB90 using default parameters. The alignments produced from this procedure were then used to build profile-HMMs using SAM's buildmodel using default parameters. These models were then calibrated and searched against the test database using SAM's hmmscore and submodel to subsequence (also termed local/local) scoring. Model to subsequence scoring (also termed global/local) calibration and searching was performed but did not perform as well on our benchmark. Likewise, SAM's various model building scripts were all tested on our benchmark and it was found that the model building script w0.5 performed the best (Data not shown). This script was used to construct the SAM profile-HMMs used in our study.

### Computational Resources

All computational experiments in this study were performed using the HHMI Janelia Farm's compute resources. The compute cluster consists of 504 nodes with 4,032 cores where each core is a 2.66 Ghz Intel Gainestown X5560 processor. All nodes have 25 GB RAM and are running RHEL 5.

## Results

### HMMERHEAD

We assessed whether we could reduce HMMER's search time by applying a series of filtering steps to the sequences in a large database (See Methods). The full Viterbi or Forward scoring algorithms would be performed on only those database sequences that passed all the filtering steps. A HMMER search performed with these filtering steps is referred to as HMMERHEAD Viterbi or HMMERHEAD Forward, according to the full scoring algorithm is performed if a sequence passes the filtering steps.

### HMMERHEAD Parameter Settings

In order to assess the effect of the various parameter settings, we randomly selected 250 benchmarking models to be used in database searches where the four HMMERHEAD parameters, θ, δ, μ, and η, were varied. Parameter effects on runtime were determined by searching the models against a non-redundant version of NCBI's NR database(NRDB90) and effects on sensitivity and specificity, as measured by minimum error rate (MER) [14] (See Methods), were determined by searching against our benchmarking database using both HMMERHEAD Viterbi and Forward scoring. While the data shown below are for the HMMERHEAD Viterbi searches, HMMERHEAD Forward closely mirrors these results. All possible combinations of the following parameter settings were tested: θ =[8.4, 7.2, 6, 4.8, 3.6], δ = [2.8, 2.4, 2, 1.6, 1.2], μ = [9.8, 8.4, 7, 5.6, 4.2], and η = [28, 24, 20, 26[12]].

To select HMMERHEAD's default parameters, we eliminated parameter settings that resulted in a greater than 0.5% increase in MER relative to HMMER's default search performance. We then selected the parameter settings that yielded the fastest runtime from the 218 different parameter combinations that passed the MER cutoff. This resulted in the HMMERHEAD Viterbi and Forward parameter settings of θ = 6, δ = 2, μ = 7, and η = 20 (×1000 bits) for HMMER models built using default settings.

**Figure 1:**
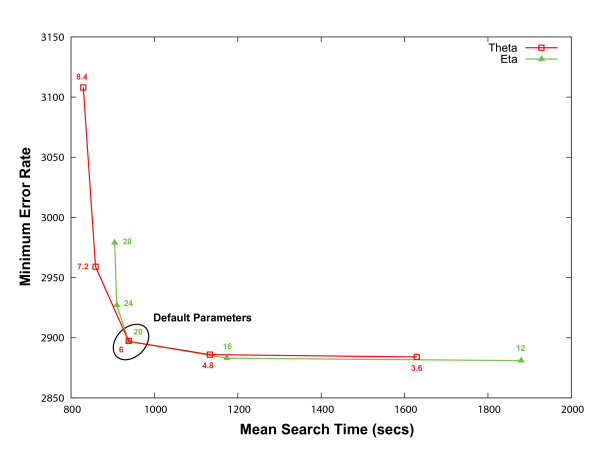
**Minimum Error Rate Relative to Mean Search Time**. 250 randomly chosen benchmarking models were used in HMMERHEAD Viterbi searches of NRDB90 and the test database utilizing a range of θ and η values. All other parameters were kept at their default value (θ = 6, δ = 2, μ = 7, and η = 20). Plotting minimum error rate versus mean search time for searches using these parameter values reveals a dramatic increase in minimum error rate, relative to a minor decrease in search time, for θ and η parameter values greater than 6 and 20, respectively. This further supports our choice of these parameter values for HMMERHEAD's default settings. The total number of true homologous pairs between those 250 models and the test benchmark was 3,617. The number of true positives identified at these parameter settings at 0 false positives is 938 or 26% of the possible true positives.

Examination of individual parameter settings revealed that the θ and η parameters had the most effect on runtime and MER (Data not shown). Plotting mean search time versus minimum error rate for the various θ and η settings reveals a strong increase in minimum error rate relative to a minor decrease in mean search time for θ and η parameter values greater than 6 and 20, respectively (Figure 1). This further supports our choice of these parameter values for HMMERHEAD's default settings.

### HMMERHEAD Search Times

In order to assess HMMERHEAD's improvement in search time, we randomly selected 500 benchmarking models and searched them against NRDB90 using Forward and Viterbi final scoring. The average time of these HMMERHEAD searches were then compared against the average default HMMER 2.5.1 search times to determine the average fold speedup (Table 1).

**Table 1:** HMMERHEAD Speed Improvement

Model	Algorithm	θ	δ	μ	η	Db Reduction	Fold Speedup
**Local**	Forward	6	2	7	20	6,805/2,313,578 = 0.3%	19,880s/957s = 20.8X

**Local**	Viterbi	6	2	7	20	6,805/2,313,578 = 0.3%	5,066s/858s = 5.9X

Average search time for 500 benchmarking models was calculated using HMMERHEAD Forward or Viterbi with several different filtering thresholds. This average time was then compared to the average search time of default HMMER 2.5.1 Forward or Viterbi, 19,880 and 5,066 seconds, respectively. HMMERHEAD Forward provides the greatest speed improvement, ~24X, due the increased average time of this scoring algorithm.

Only 6,805 comparisons, out of 2,313,578, passed the HMMERHEAD filtering steps. This reduced the effective database size that the full scoring algorithms were performed on by 99.7%. HMMERHEAD Forward provides an approximate 20-fold speedup versus default HMMER 2.5.1 Forward scoring. HMMERHEAD Viterbi is approximately 6-fold faster. HMMER's Forward implementation is approximately 4-fold slower than Viterbi and explains the difference in HMMERHEAD speedup between algorithms.

### Remote Homology Detection of HMMERHEAD Versus HMMER 2.5.1

Since HMMERHEAD's speed gain is due to the utilization of search heuristics, we determined the cost of these heuristics on sensitivity. Using our remote protein homology benchmark, we compared the sensitivity and specificity of HMMERHEAD versus HMMER 2.5.1 for Forward and Viterbi scoring. In addition, we included the performance of WU-BLASTP and the WU-BLAST Family Pairwise Search method. This method has been previously described and consists of comparing a 'family' of sequences in a pairwise fashion to a database [16]. The E-values of any shared targets are then combined (See Methods) to determine the similarity between the sequence family and database targets.

**Figure 2:**
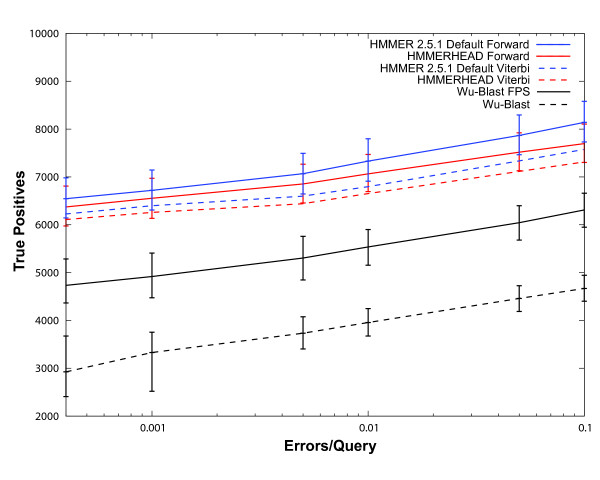
**Remote Homology Detection of HMMERHEAD and HMMER 2.5.1 Forward and Viterbi**. Each of the 2,521 benchmarking models was scored against the test database using either HMMER 2.5.1 or HMMERHEAD with Forward or Viterbi scoring. The results of the searches were combined and scored. This procedure was then repeated for each of the 1000 bootstrapping replicate test sets. The average number of true positives was plotted versus errors per query. Minimum and maximum numbers of true positives from the replicates are shown as error bars. HMMERHEAD Forward (Red) performance is shown relative to default HMMER 2.5.1 Forward (Blue), WU-BLAST (Black dashed), and WU-BLAST Family Pairwise Search (Black). Default HMMERHEAD Forward detects an average of 269 fewer true positives than default HMMER 2.5.1 Forward and detects significantly more true positives than either pairwise sequence comparison method. HMMERHEAD Viterbi (Red dashed) performance is shown relative to default HMMER 2.5.1 Viterbi (Blue dashed). Default HMMERHEAD Viterbi detects an average of 173 fewer true positives than default HMMER 2.5.1 Viterbi and again outperforms either pairwise comparison method. The total number of true homologous pairs between the 2,521 models and the test database is 39,733, and thus 8,000 true positives correspond to identifying 20% of the homologs.

On our benchmark, HMMERHEAD Forward detects an average of 269 fewer true positives than HMMER 2.5.1 Forward across the measured specificity range (Figure 2). While this is a statistically significant amount, as determined using bootstrapping and a 95% confidence limit, this represents only a 4% loss in the number of true homologs identified by HMMER 2.5.1 Forward. HMMERHEAD Viterbi detects an average of 173 fewer true positives than HMMER 2.5.1 Viterbi on our benchmark (Figure 2). Again, this is a statistically significant amount but represents only a 2% loss in the number of true homologs identified by HMMER 2.5.1 Viterbi. Both pairwise comparison methods were significantly outperformed by HMMER 2.5.1 with and without HMMERHEAD. The HMMERHEAD speed gains of 20X and 6X compared to the sensitivity losses of 4% and 2% were deemed to be acceptable tradeoffs.

### JackHMMER

We implemented an iterative Profile hidden Markov model procedure that took advantage of our development of HMMERHEAD. This allowed us to iteratively search over a large sequence database instead of using the subdatabase approach utilized by other iterative profile-HMM implementations [4,5]. We refer to our iterative search procedure as JackHMMER.

### JackHMMER Performance

The individual benchmarking sequences were used in iterative searches against NRDB90 using JackHMMER, SAM's target2k, and NCBI's PSI-BLAST. The final models created by these iterative methods were then searched against the benchmarking database to determine the sensitivity and specificity of each method.

**Figure 3:**
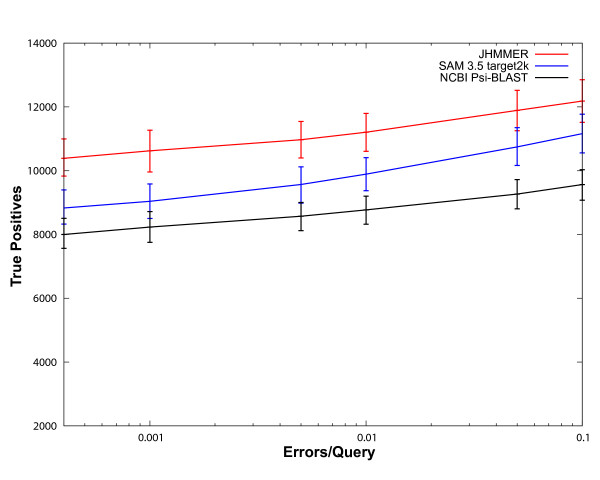
**Performance of Iterative Methods**. The individual 2,521 benchmarking sequences were used to iteratively search a non-redundant version of NCBI's NR database. The iterative models created from this process were then scored against the test database. The results of the searches were combined and scored. This procedure was then repeated for each of the 1000 bootstrapping replicate testsets. The average number of true positives was plotted versus errors per query. Minimum and maximums numbers of true positives from the replicates are shown as error bars. JackHMMER (Red) detects an average of 1,337 more homologs than SAM 3.5's target2k (Blue) and an average of 2,476 more homologs than NCBI's PSI-BLAST (Black) across the errors per query range. This represents an increase of 14% and 28% in remote protein homologs detected. As before, the total number of true homologous pairs between the 2,521 models and the test database is 39,733, and thus 12,000 true positives correspond to identifying 30% of the homologs.

On our benchmark, JackHMMER is able to detect an average of 1,337 more remote protein homologs than target2k and an average of 2,476 more homologs than PSI-BLAST across the measured specificity range (Figure 3). Thus, on our benchmark, JackHMMER is able to detect 14% more true homologs than target2k and 28% more than PSI-BLAST. Using bootstrapping and a 95% confidence limit, these increases in homolog detection are statistically significant.

## Discussion and Conclusions

In this study, we have implemented several heuristic database filtering steps, HMMERHEAD, in an effort to decrease the time required to score profile hidden Markov models against a large protein sequence database. Utilizing four filtering steps, we have managed to decrease the search time by 6 or 20-fold relative to using traditional Viterbi or Forward scoring. This decrease in search time is achieved with only a 2% or 4% loss in true homologs identified on our benchmark. This study demonstrates such heuristic database filtering steps can be successfully utilized to speedup scoring profile hidden Markov models against large sequence databases with a minimal loss in sensitivity.

Additionally, we have developed an iterative profile-HMM approach, JackHMMER. JackHMMER takes an initial query sequence and is capable of iteratively searching over a large sequence database. Due to the length of time utilized by full profile-HMM scoring algorithms, previous published iterative profile-HMM approaches required the creation of a subdatabase using a less sensitive pairwise sequence comparison method. We have leveraged HMMERHEAD's speed gains to eliminate this step from our iterative search method. Utilizing our protein homology benchmark, JackHMMER detects 28% and 14% more remote protein homologs than NCBI's PSI-BLAST and SAM's iterative profile-HMM method, target2k, which are statistically significant improvements.

## Availability

HMMER 2.5.1, which contains both HMMERHEAD and JackHMMER, is available for download from http://selab.janelia.org/publications/Johnson10/hmmer2.5.1.tar.gz.

The multiple sequence alignments, test database, and Perl scripts used in our benchmark are available as a compressed tar archive at http://selab.janelia.org/publications/Johnson10/scripts_aln_dbs.tar.gz.

## Authors' contributions

LSJ contributed to the design and testing of the HMMERHEAD algorithm, designed and tested the JackHMMER algorithm, and drafted the manuscript. EP conceived of and contributed to the design and testing of the HMMERHEAD algorithm. SRE oversaw the design of this study. All authors read and approved the final manuscript.

## References

[B1] HMMER: biosequence analysis using profile hidden Markov models http://hmmer.org/

[B2] Altschul SF Gish W Miller W Myers EW Lipman DJ Basic local alignment search tool J Mol Biol 1990 3 403 410 10.1016/S0022-2836(05)80360-2 2231712

[B3] Altschul SF Madden TL Schaffer AA Zhang J Zhang Z Miller W Lipman DJ Gapped BLAST and PSI-BLAST: a new generation of protein database search programs Nucleic Acids Res 1997 25 17 3389 3402 10.1093/nar/25.17.3389 9254694 PMC146917

[B4] Karplus K Barrett C Hughey R Hidden Markov models for detecting remote protein homologies Bioinformatics 1998 14 10 846 856 10.1093/bioinformatics/14.10.846 9927713

[B5] Scheeff ED Bourne PE Application of protein structure alignments to iterated hidden Markov model protocols for structure prediction BMC Bioinformatics 2006 7 410 10.1186/1471-2105-7-410 16970830 PMC1622756

[B6] Schaffer AA Aravind L Madden TL Shavirin S Spouge JL Wolf YI Koonin EV Altschul SF Improving the accuracy of PSI-BLAST protein database searches with composition-based statistics and other refinements Nucleic Acids Res 2001 29 2994 3005 10.1093/nar/29.14.2994 11452024 PMC55814

[B7] Holm L Sander C Removing near-neighbour redundancy from large protein sequence collections Bioinformatics 1998 14 5 423 429 10.1093/bioinformatics/14.5.423 9682055

[B8] Brenner SE Chothia C Hubbard TJ Assessing sequence comparison methods with reliable structurally identified distant evolutionary relationships Proc Natl Acad Sci USA 1998 95 11 6073 6078 10.1073/pnas.95.11.6073 9600919 PMC27587

[B9] Lo Conte L Ailey B Hubbard TJ Brenner SE Murzin AG Chothia C SCOP: a structural classification of proteins database Nucleic Acids Res 2000 28 1 257 259 10.1093/nar/28.1.257 10592240 PMC102479

[B10] Finn RD Tate J Mistry J Coggill PC Sammut SJ Hotz HR Ceric G Forslund K Eddy SR Sonnhammer EL Bateman A The Pfam protein families database Nucleic Acids Res 2008 36 D281 8 10.1093/nar/gkm960 18039703 PMC2238907

[B11] Brenner SE Koehl P Levitt M The ASTRAL compendium for protein structure and sequence analysis Nucleic Acids Res 2000 28 254 256 10.1093/nar/28.1.254 10592239 PMC102434

[B12] Chandonia JM Walker NS Lo Conte L Koehl P Levitt M Brenner SE ASTRAL compendium enhancements Nucleic Acids Res 2002 30 260 263 10.1093/nar/30.1.260 11752310 PMC99063

[B13] Madera M Gough J A comparison of profile hidden Markov model procedures for remote homology detection Nucleic Acids Res 2002 30 19 4321 4328 10.1093/nar/gkf544 12364612 PMC140544

[B14] Pearson WR Comparison of methods for searching protein sequence databases Protein Sci 1995 4 1145 1160 10.1002/pro.5560040613 7549879 PMC2143149

[B15] Price GA Crooks GE Green RE Brenner SE Statistical evaluation of pairwise protein sequence comparison with the Bayesian bootstrap Bioinformatics 2005 21 3824 3831 10.1093/bioinformatics/bti627 16105900

[B16] Grundy WN Homology detection via family pairwise search J Comput Biol 1998 5 3 479 491 10.1089/cmb.1998.5.479 9773344

